# PGC-1α attenuates hydrogen peroxide-induced apoptotic cell death by upregulating Nrf-2 via GSK3β inactivation mediated by activated p38 in HK-2 Cells

**DOI:** 10.1038/s41598-017-04593-w

**Published:** 2017-06-28

**Authors:** Hoon-In Choi, Hye-Jin Kim, Jung-Sun Park, In-Jin Kim, Eun Hui Bae, Seong Kwon Ma, Soo Wan Kim

**Affiliations:** Department of Internal Medicine, Chonnam National University Medical School, Gwangju, Republic of Korea

## Abstract

Ischemia/reperfusion injury triggers acute kidney injury (AKI) by aggravating oxidative stress mediated mitochondria dysfunction. The peroxisome proliferator-activated receptor gamma coactivator 1α (PGC-1α) is a master player that regulates mitochondrial biogenesis and the antioxidant response. We postulated that PGC-1α functions as cytoprotective effector in renal cells and that its regulation mechanism is coordinated by nuclear factor erythroid 2-related factor 2 (Nrf-2). In this study, to understand the effect and molecular mechanisms of PGC-1α, we developed an empty vector or PGC-1α-overexpressing stable cell lines in HK-2 cells (Mock or PGC-1α stable cells). PGC-1α overexpression increased the viability of cells affected by H_2_O_2_ mediated injury, protected against H_2_O_2_-mediated apoptotic events and inhibited reactive oxygen species accumulation in the cytosol and mitochondria as compared to that in Mock cells. The cytoprotective effect of PGC-1α was related to Nrf-2 upregulation, which was counteracted by Nrf-2-specific knockdown. Using inhibitor of p38, we found that regulation of the p38/glycogen synthase kinase 3β (GSK3β)/Nrf-2 axis was involved in the protective effects of PGC-1α. Taken together, we suggest that PGC-1α protects human renal tubule cells from H_2_O_2_-mediated apoptotic injury by upregulating Nrf-2 via GSK3β inactivation mediated by activated p38.

## Introduction

Acute kidney injury (AKI), defined as a rapid decline of renal function, is a common complication in hospitalized patients and leads to increased morbidity and mortality. Along with nephrotoxin injury and sepsis, renal ischemia/reperfusion (I/R) injury is one of the main causes of AKI^1, 2^. Mitochondrial dysfunction, such as release of cytochrome *C*, mitochondrial-permeability transition (MPT) activation, and caspase activation, triggers I/R-induced apoptosis processes^3–5^. Multiple studies have demonstrated that I/R injury is associated with increased levels of reactive oxygen species (ROS), which originates in the mitochondria^6, 7^. Pre-treatment with the mitochondria-targeted antioxidants MitoQ and Mito-CP prevents cisplatin- and I/R-induced oxidative stress and tubular apoptosis in the kidney and liver^8, 9^. Therefore, treatment strategies that target the mitochondrial functions may be of interest to prevent ROS-mediated AKI.

Peroxisome proliferator-activated receptor γ coactivator 1α (PGC-1α) is an inducible transcription coactivator that is involved in adaptive thermogenesis, skeletal muscle fiber type switching, glucose/fatty acid metabolism, and heart development via its ability to promote mitochondrial energy metabolism^10–13^. Mitochondrial dysfunction and impaired PGC-1α are intimately related to various diseases, such as obesity, type 2 diabetes, and cardiomyopathy^14^. Moreover, there have been many reports on the beneficial effects of PGC-1α as a master regulatory protein of mitochondrial function. Recently, a study on urine metabolomics reported that mitochondrial dysfunction in diabetic kidney disease is associated with reduced PGC-1α mRNA and mtDNA^15^. During cisplatin-induced AKI, down-regulation of PGC-1α mRNA in proximal tubule cells leads to acute tubular necrosis caused by inhibition of mitochondrial fatty acid oxidation^16^. Moreover, treatment with PPARγ agonists promotes PGC-1 α induction, and their effects ameliorate AKI^17^. However, the effect of PGC-1α in apoptotic cellular injury is controversial. Some studies have shown that a PPARγ-dependent down-regulation of PGC-1α promotes cancer growth and progressions in several cancers^18–21^. Further, transient expression of PGC-1α in mouse cardiac-derived H9c2 cells increases cell death after ischemia-reoxygenation injury^22^. Although mitochondria dysfunction is a major characteristic of a diverse range of diseases, cell fate is differently determined by altered gene expression patterns in a cell type- or tissue type-specific manner.

Nuclear factor erythroid 2-related factor 2 (Nrf-2; *NFE2L2*) plays a central role not only in overall cellular redox homeostasis by regulating the coordinated induction of cytoprotective genes^23^ but also in enhancing the structural and functional integrity of mitochondria under stress conditions via relationship with various proteins^24^. Bardoxolone methyl, a first-in-class oral Nrf-2 agonist, has been shown to improve kidney function in diabetic nephropathy patients with transcriptional expression of network genes (like as PGC-1α, nuclear respiratory factor-1) that are linked with mitochondrial function^25^. Further, Nrf-2 null mice were found to be markedly sensitized to cisplatin-induced AKI^26^. It could be assumed that PGC-1α and Nrf-2 may be part of the same signaling pathway involved in the maintenance of both cellular redox homeostasis and mitochondrial homeostasis. Recently, some studies reported that PGC-1α is involved in the regulation of the Nrf-2 expression and activity^27, 28^, and direct interaction between PGC-1α and Nrf-2 was also identified by protein-protein interaction^29^. However, the molecular mechanisms that interconnect the functional relation between PGC-1α and Nrf-2 are not still completely understood.

In this study, we evaluated whether the expression of PGC-1α is involved in I/R induced kidney injury and H_2_O_2_-treated human proximal epithelial tubule (HK-2) cells. We also investigated whether PGC-1α overexpression has a beneficial or maladaptive effect against H_2_O_2_-mediated apoptosis and ROS accumulation by using stable PGC-1α-overexpressing HK-2 cells. We analyzed whether the PGC-1α/Nrf-2 has a cytoprotective effect on H_2_O_2_-treated HK-2 cells, including Nrf-2 mediated antioxidant response element (ARE) activation, reduction of apoptotic signal activation, and ROS accumulation. We hypothesized that activation of p38 and sequential inactivation of glycogen synthase kinase 3β (GSK3β), which is mediated by PGC-1α overexpression, would be one of molecular mechanisms for effective PGC-1α/Nrf-2 axis. Therefore, we assumed that PGC-1α-dependent Nrf-2 upregulation may be a crucial part for the protective effect against H_2_O_2_-mediated apoptosis in HK-2 cells.

## Results

### PGC-1α was downregulated in the I/R-injured kidney, as well as in H_2_O_2_ treated HK-2 cells

**Figure 1:**
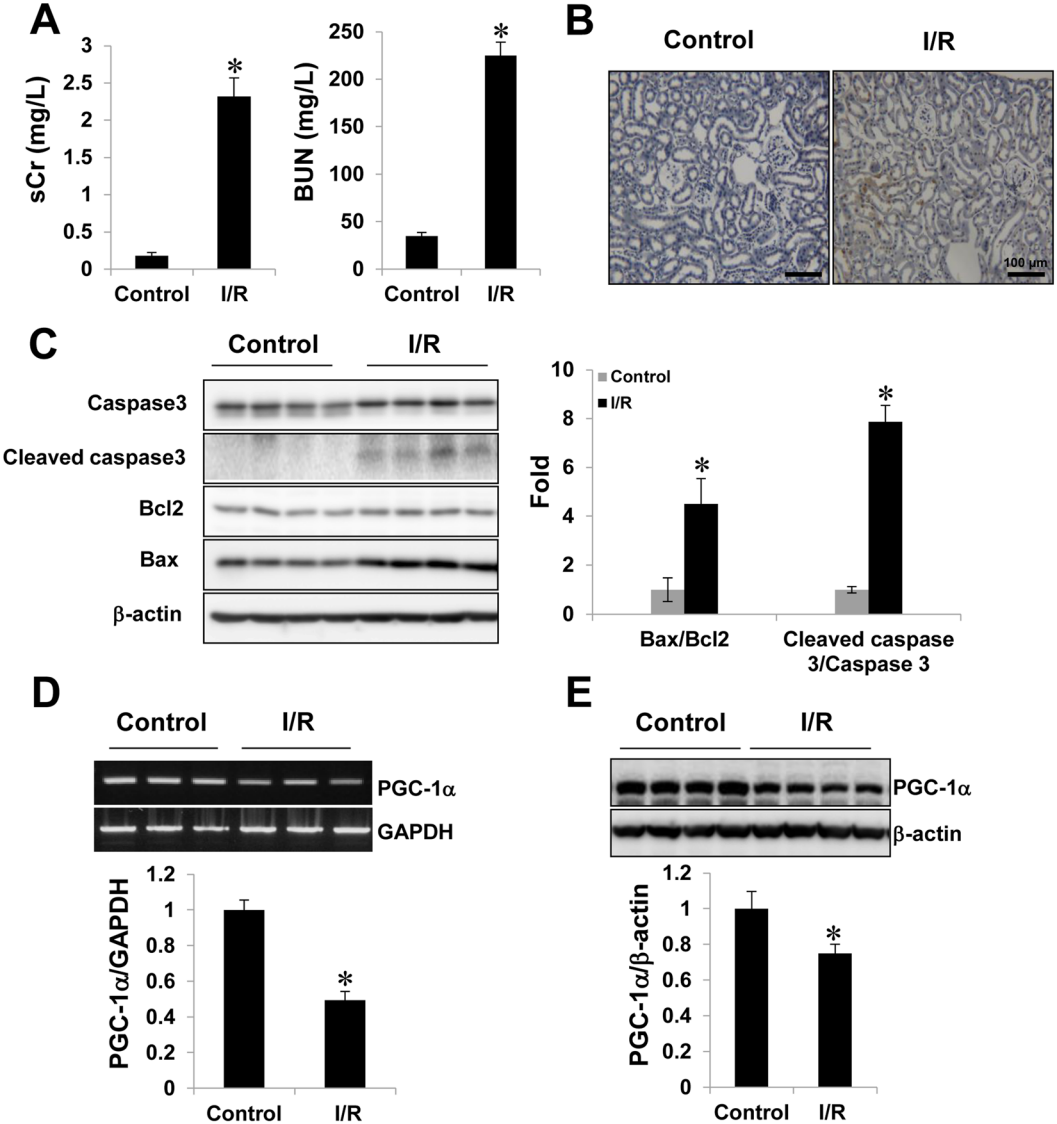
Downregulation of PGC-1α in I/R-induced kidney injury. (**A**) Plasma creatinin and blood urea nitrogen (BUN) levels of control vs. I/R-induced kidney injury in mice. (**B**) Terminal deoxynucleotidyl transferase-mediated dUTP nick end-labeling (TUNEL) staining of the kidney. Magnification at x100, Bar = 100 μm. (**C**) I/R-induced kidney injury was assessed by evaluating the expression of apoptotic proteins (ratio of Bax to Bcl2 and cleaved caspase 3 to caspase 3). (**D**) The mRNA expression of PGC-1α in control mice vs. those that underwent I/R-induced kidney injury. (**E**) Protein expression of PGC-1α in control mice vs. those that underwent I/R-induced kidney injury. The bar graph shows the indicated target expression measured by densitometry. GAPDH and β-actin levels were analyzed as internal controls. Full-length blots of each tested protein are reported in Supplementary Figure S1. *p < 0.05 control vs. I/R group.

**Figure 2:**
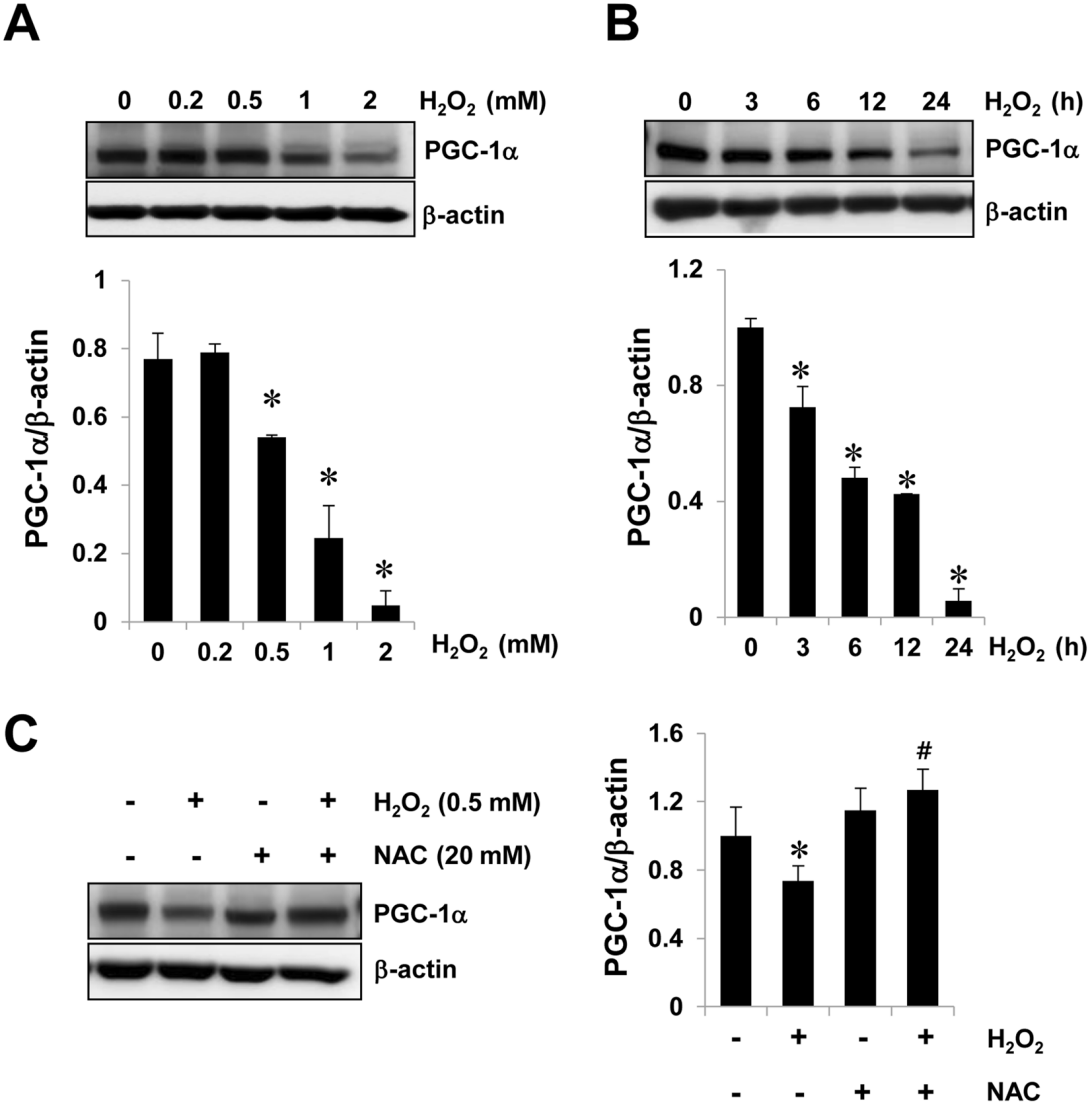
Downregulation of PGC-1α in H_2_O_2_-treated HK-2 cells. To mimic I/R-induced oxidative stress in an *in vitro* system, we treated with H_2_O_2_ in HK-2 cells. (**A**) Dose-dependent PGC-1α expression. HK-2 cells were treated with an indicated H_2_O_2_ concentration (0, 0.2, 0.5, 1, and 2) for 6 h. (**B**) Time-dependent PGC-1α expression. HK-2 cells were treated with 0.5 mM H_2_O_2_ for an indicated time (0, 3, 6, 12, and 24 h). (**C**) To assess the effect ROS in H_2_O_2_ induced PGC-1α downregulation, cells were incubated for 6 h with 0.5 mM H_2_O_2_ in the presence or absence of 20 mM NAC. The bar graph shows the relative protein expression of PGC-1α measured by densitometry. β-actin levels were analyzed as internal controls. Full-length blots of each tested protein are reported in Supplementary Figure S2. Error bars denote the mean ± S.D. of triplicate samples. *p < 0.05 H_2_O_2_-treated vs. H_2_O_2_-untreated. ^#^ *p* < 0.05 H_2_O_2_-treated in the presence of NAC vs. H_2_O_2_-treated in the absence of NAC.

To investigate the involvement of PGC-1α in I/R-induced AKI, we analyzed the PGC-1α expression pattern in I/R induced mouse model. Groups that were subjected to renal I/R (n = 4) showed marked deterioration of renal functional parameters along with significant increase in the plasma creatinine level (sCr) and blood urea nitrogen (BUN), as compared to the finding for the controls (n = 4; Fig. 1A). We then performed a terminal deoxynucleotidyl transferase dUTP nick end labeling (TUNEL) assay to determine the degree of renal tubular apoptosis in I/R-injured kidney. We found an increased number of tubular epithelial cells with TUNEL-positive nuclei in I/R-injured kidney (Fig. 1B). The levels of apoptotic proteins, for example, the Bax to Bcl2 ratio and the cleaved caspase 3 to caspase 3 ratio, also increased in the I/R group (Fig. 1C). The mRNA level and protein level of PGC-1α were lower in the I/R-injured kidney group as compared to those in the control group (Fig. 1D,E). We assessed the effect of PGC-1α in HK-2 cells under oxidative stress condition. To mimic I/R stress in HK-2 cells, we treated them with H_2_O_2_, which is the main culprit in the pathogenesis of I/R injury^30^. The PGC-1α expression level in H_2_O_2_-treated HK-2 cells was gradually decreased under condition of concentrations (0.5 mM) and duration (3 h) of treatment with H_2_O_2_ (Fig. 2A,B). We further assessed whether H_2_O_2_-induced PGC-1α downregulation was dependent on ROS level by H_2_O_2_ treatment and, if so, whether PGC-1α downregulation could be inhibited by removing ROS using the well-known chemical antioxidant N-acetyl-L-cysteine (NAC). The level of PGC-1α expression, which was downregulated by H_2_O_2_ was restored by 20 mM of NAC (Fig. 2C).

### PGC-1α overexpression protected cells from H_2_O_2_-mediated injury

**Figure 3:**
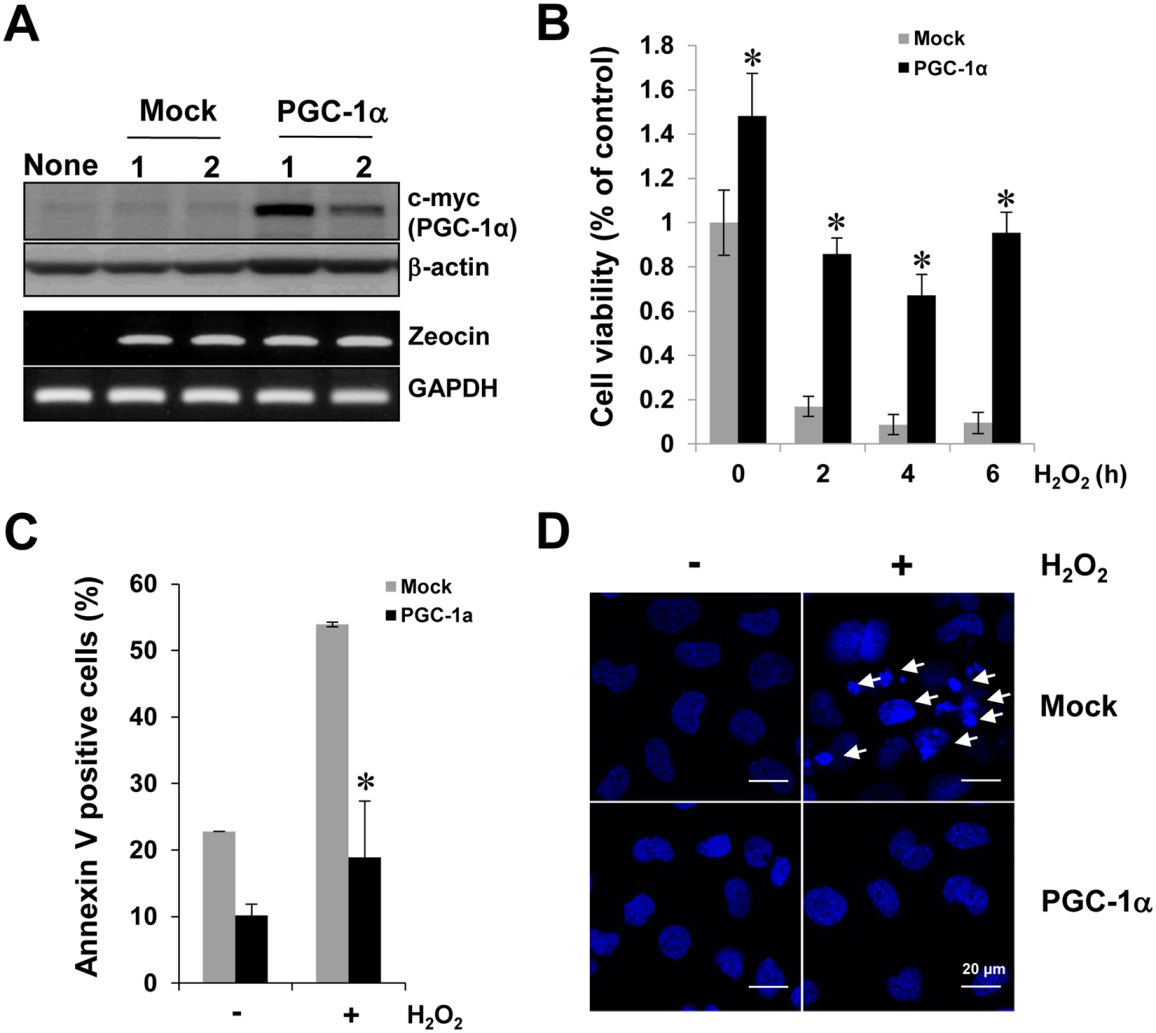
Protective effect of PGC-1α in H_2_O_2_-mediated cellular injury. To verify the role of PGC-1α in H_2_O_2_-mediated cellular injury, we developed stable cell lines over-expressing PGC-1α in HK-2 cells, as mentioned in the Materials and Methods section. (**A**) Expression of PGC-1α in Mock and PGC-1α stable cells. The protein expression (upper panel) of c-terminal c-Myc tagged PGC-1α was assessed with anti-c-Myc and stable cells were selected via the confirmation of zeocine expression (upper panel), which was contained in the backbone plasmid, *pCDNA4*. Full-length blots of each tested protein are reported in Supplementary Figure S3. (**B**) Cell viability of Mock and PGC-1α-stable cells. Stable cells were treated with 0.5 mM H_2_O_2_ for an indicated time (0, 2, 4, and 6 h). Cell viability was evaluated by the MTT method. (**C**) Quantification of apoptotic cells. Mock and PGC-1α cells were treated with 0.5 mM H_2_O_2_ for 4 h. Apoptotic cell numbers were measured by counting Annexin V positive cells using a FACSCalibur^TM^ flow cytometry. (**D**) Apoptotic body formation in H_2_O_2_-treated Mock and PGC-1α stable cells. Apoptotic body formation (arrows), as an indicator of apoptosis, was determined by DAPI staining then photographing cells under fluorescence microscopy as described in the Materials and Methods. Image was magnified at x800, Bar = 20 μm. Error bars denote the mean ± S.D. of triplicate samples. *p < 0.05 Mock vs. PGC-1α.

To examine the physiological effect of PGC-1α in proximal tubule cells, we stably transfected HK-2 cells with an empty vector (Mock) or a plasmid encoding human *PGC-1α* (PGC-1α) (Fig. 3A). Expression of c-terminal c-Myc tagged PGC-1α was assessed with anti-c-Myc antibody. Stable cells clone were selected via confirmation of expression of zeocine, which was present in the backbone plasmid, *pCDNA4*. Evaluation of Mock and PGC-1α stable cells following H_2_O_2_-mediated injury revealed increase in cell viability (Fig. 3B) and decrease in the number of Annexin-V-positive cells (Fig. 3C) or in apoptotic body formation (Fig. 3D) in PGC-1α stable cells. This result showed that PGC-1α overexpression alleviates H_2_O_2_-mediated apoptotic and necrotic cell death.

### PGC-1α had an anti-apoptotic effect

**Figure 4:**
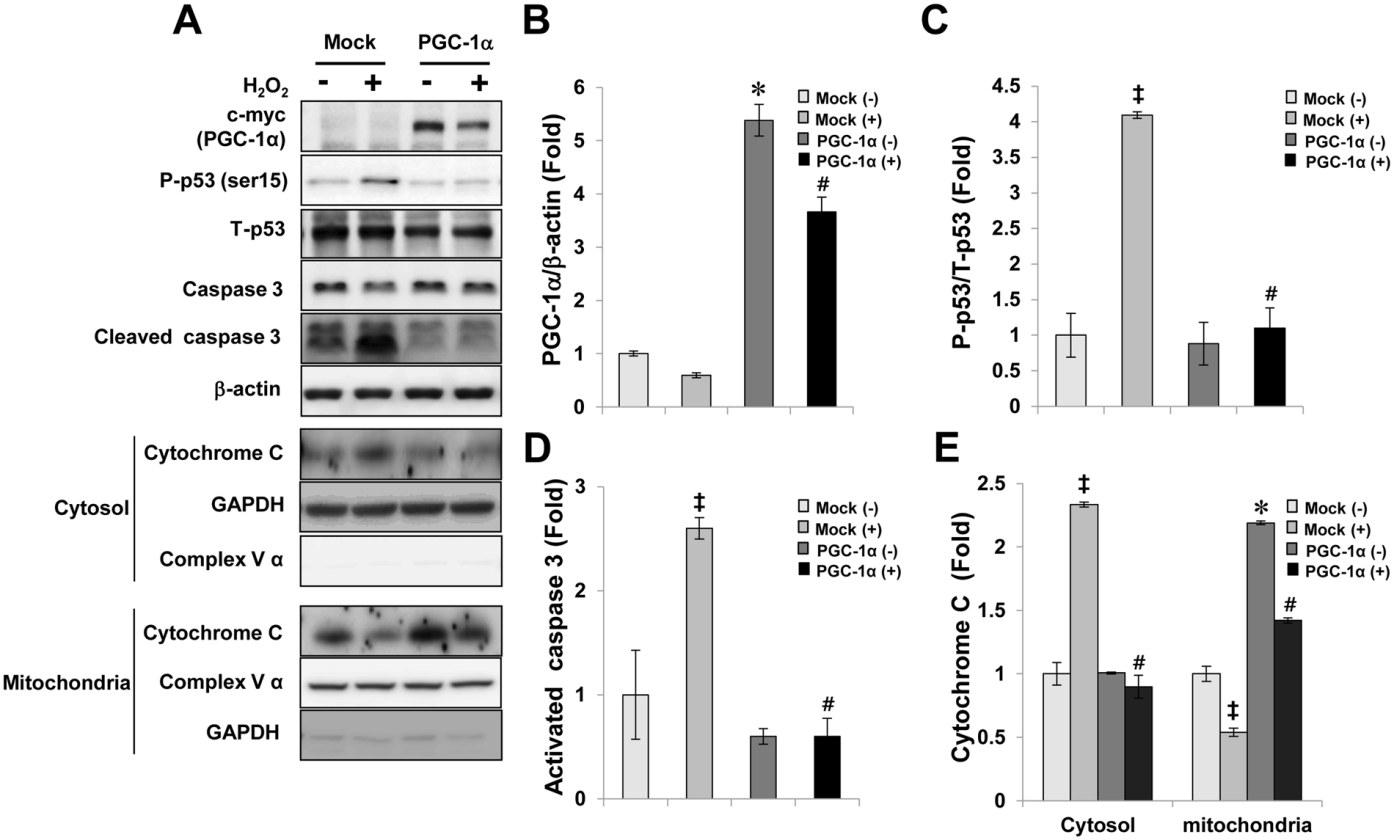
Anti-apoptotic effect of PGC-1α. Stable cells were treated with 0.5 mM H_2_O_2_ for 6 h. (**A**) The expression bands of apoptotic proteins in Mock and PGC-1α-stable cells were compared via western blotting. Each bar graph represents the expression of PGC-1α (**B**), ratio of phosphorylated p53 at Ser 15 to total p53 (**C**), the level of activated caspase 3 (ratio of cleaved caspase 3 to caspase 3 (**D**), and the level of cytochrome C release from mitochondria to cytosol (**E**). β-actin levels were analyzed as internal controls. GAPDH and complex V α were used as internal controls in cytosol and in mitochondria fraction, respectively. Full-length blots of each tested protein are reported in Supplementary Figure S4. Error bars denote the mean ± S.D. of triplicate samples. *p < 0.05 H_2_O_2_-untreated Mock vs. PGC-1α; ^#^ *p* < 0.05; H_2_O_2_-treated Mock vs. PGC-1α; ^‡^ *p* < 0.05; H_2_O_2_-treated Mock vs. H_2_O_2_-untreated Mock.

To verify the protective effects of PGC-1α in injured proximal tubule cells, the expression and activation of pro-apoptotic proteins was evaluated in H_2_O_2_-treated Mock and PGC-1α stable cells (Fig. 4). Although PGC-1α expression was lower in H_2_O_2_-treated PGC-1α stable cells than in H_2_O_2_-untreated PGC-1α stable cells, PGC-1α expression was higher in PGC-1α stable cells than in Mock cells (Fig. 4A). The phosphorylation of p53 at Ser 15, which is involved in stabilization and mitochondrial accumulation of p53, were lesser in H_2_O_2_-treated PGC-1α stable cells than in Mock cells (Fig. 4A,C). Further, the level of activated caspase 3, assessed using the cleaved caspase 3 to caspase 3 ratio, markedly reduced in H_2_O_2_-treated PGC-1α stable cells (Fig. 4A,D). Release of mitochondrial cytochrome *C* to the cytosol, which resulted in activation of caspase 3, was also lesser in H_2_O_2_-treated PGC-1α stable cells than in Mock cells (Fig. 4A,E).

### PGC-1α had an anti-oxidative effect

**Figure 5:**
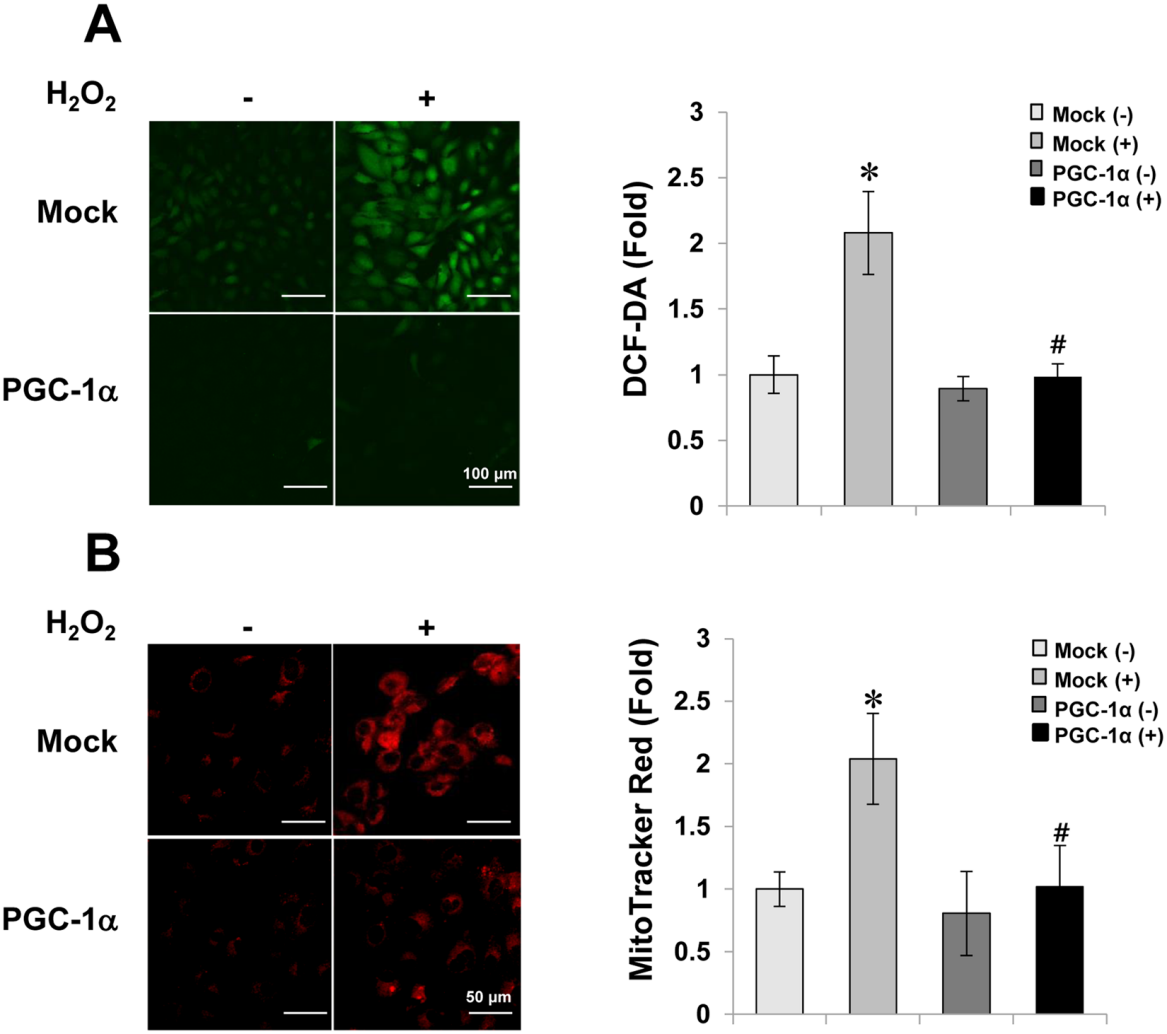
Reduction of intracellular ROS by PGC-1α. To check the efficiency of PGC-1α overexpression in reducing ROS, stable cells were plated onto four well-cell culture slides (5 × 10^4^/well) and treated with 0.5 mM H_2_O_2_ for 30 min. Cytosolic (magnification at x200, Bar = 100 μm) (**A**) and mitochondrial ROS (magnification at x400, Bar = 50 μm) (**B**) were labeled using CM-H_2_DCFDA or CM-H_2_XROS probes, respectively. Images were immediately visualized using confocal microscopy on a laser scanning microscope (LSM 510; Carl Zeiss), and analyzed using a LMS 5 browser imaging software. Error bars denote the mean ± S.D. of triplicate samples. *p < 0.05 H_2_O_2_-untreated vs. H_2_O_2_-treated in Mock; ^#^ *p* < 0.05; H_2_O_2_-treated Mock vs. PGC-1α.

Many earlier studies showed that PGC-1α suppresses ROS production through the induction of ROS-detoxifying enzymes^31, 32^. Therefore, we confirmed whether intracellular ROS in cytosol or in mitochondria could be regulated by PGC-1α overexpression in our system using a selective ROS probe, CM-H_2_DCF-DA or MitoTracker Red CM-H_2_XRos, respectively. In PGC-1α stable cells, ROS levels were noticeably lower in the mitochondria as well as the cytosol, as compared to levels in Mock cells (Fig. 5).

### Upregulation of Nrf-2 expression by PGC-1α was involved in the cytoprotective effects of PGC-1α

**Figure 6:**
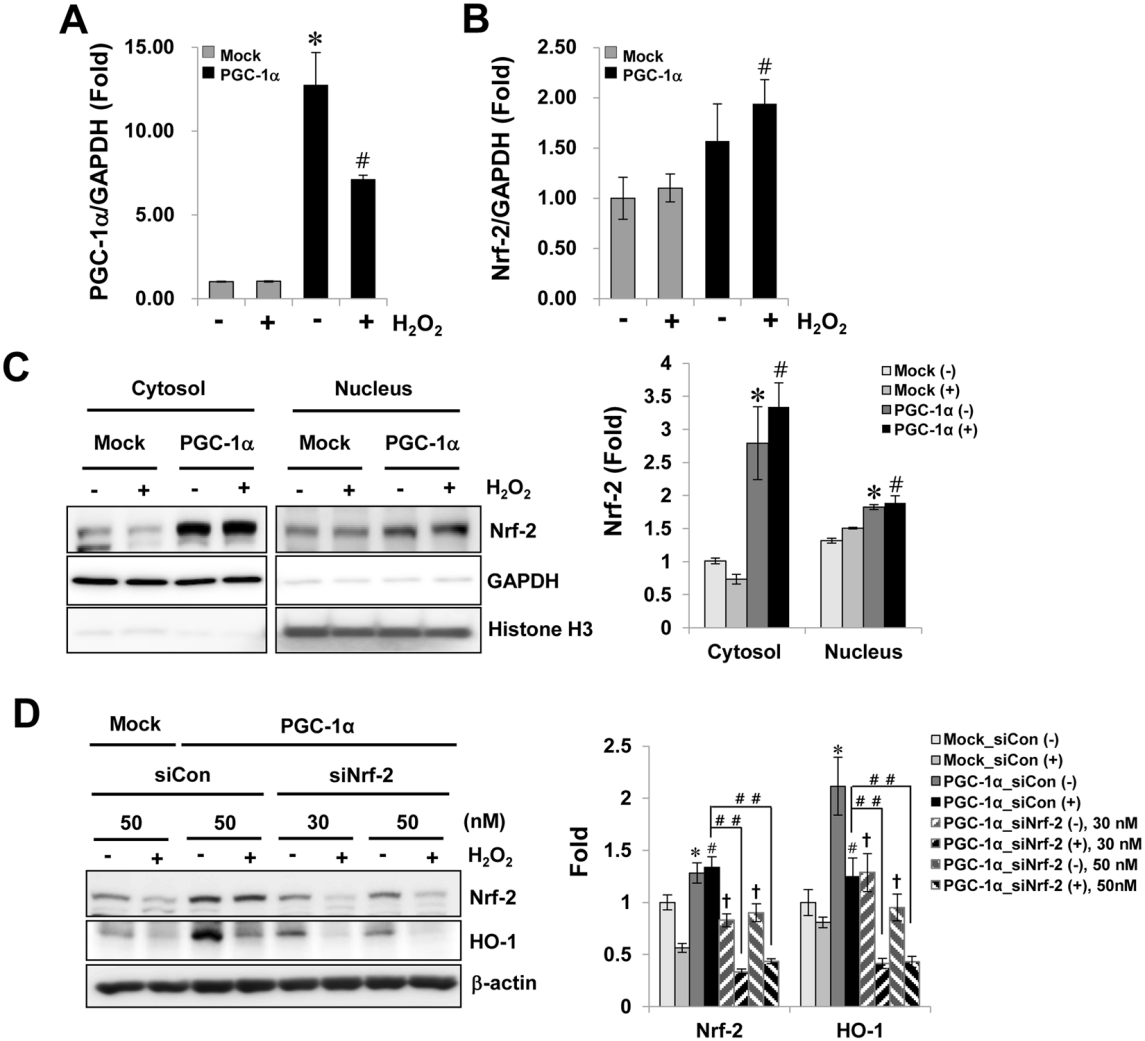
Upregulation of Nrf-2 by PGC-1α overexpression. The change in Nrf-2 mRNA and protein levels by PGC-1α overexpression was evaluated in stable cells. Cells were treated with 0.5 mM H_2_O_2_ for 6 h. The mRNA levels of PGC-1α (**A**) and Nrf-2 (**B**) were analyzed by real-time PCR with each PCR primer pair. GAPDH was analyzed as an internal control. (**C**) Upregulation of Nrf-2 protein in cytosol and nucleus fraction by PGC-1α overexpression. (**D**) Regulation of Nrf-2 mediated HO-1 expression by PGC-1α. To assess the involvement between PGC-1α expression and Nrf-2 mediated HO-1 (as a one of well-known downstream molecules activated by Nrf-2) expression for protective effect of PGC-1α, PGC-1α stable cells were incubated with Nrf-2 specific siRNA (30 and 50 nM), and then, after 2 days, cells were treated with 0.5 mM H_2_O_2_ for 6 h. The protein level of Nrf-2 and HO-1 were determined by immunoblotting with specific antibodies. Relative protein level was expressed as fold increases normalized to the untreated Mock cells. β-actin levels were analyzed as internal controls. Full-length blots of each tested protein are reported in Supplementary Figure S5. Error bars denote the mean ± S.D. of triplicate samples. *p < 0.05 H_2_O_2_-untreated Mock vs. PGC-1α; ^#^ *p* < 0.05; H_2_O_2_-treated Mock vs. PGC-1α; ^##^ *p* < 0.05; H_2_O_2_-treated siCon vs. siNrf-2 in PGC-1α stable cells; ^†^ *p* < 0.05; H_2_O_2_-untreated siCon vs. siNrf-2 in PGC-1α stable cells.

To further analyze the protective mechanism of the PGC-1α-associated anti-apoptotic effect and anti-oxidative effect, we studied the expression of Nrf-2, which is involved in the coordinated induction of genes that encode many stress-responsive and cytoprotective enzymes and related proteins. The Nrf-2 mRNA levels increased in H_2_O_2_-treated PGC-1α cells (Fig. 6A,B). The Nrf-2 protein level also increased following PGC-1α overexpression both in the cytosol and the nucleus (Fig. 6C). Moreover, the expression of heme oxygenase-1 (HO-1), known as a downstream target molecules of Nrf-2, also increased in consistent to Nrf-2 upregulation in PGC-1α stable cells, its expression in PGC-1α stable cells decreased on Nrf-2 specific siRNA knockdown in PGC-1α stable cells (Fig. 6D).

**Figure 7:**
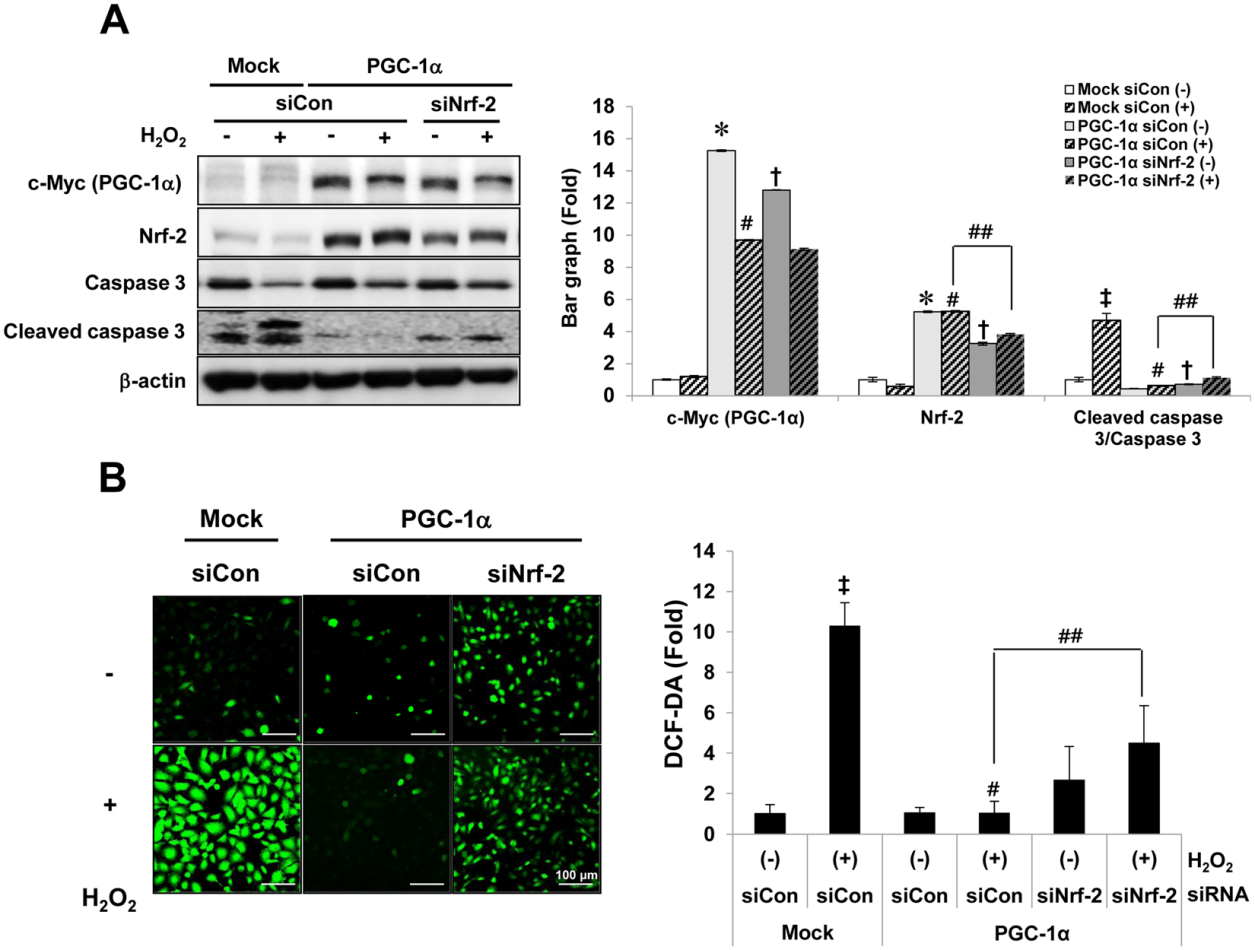
Nrf-2 specific-protective effects by PGC-1α. To prove the dependence of Nrf-2 in anti-apoptotic and anti-oxidative effect of PGC-1α, the level of activated caspase 3. Full-length blots of each tested protein are reported in Supplementary Figure S6 (**A**) and the level of DCF fluorescence (**B**) were assessed in Nrf-2 suppressed PGC-1α cells under H_2_O_2_ treatment, as earlier mentioned. Magnification at x200, Bar = 100 μm. Relative protein level and ROS level were expressed as fold normalized to the untreated Mock cells. β-actin levels were analyzed as internal controls. Error bars denote the mean ± S.D. of triplicate samples. *p < 0.05 H_2_O_2_-untreated Mock vs. PGC-1α; ^‡^ *p* < 0.05 H_2_O_2_-untreated vs. H_2_O_2_-treated Mock; ^#^ *p* < 0.05; H_2_O_2_-treated Mock vs. PGC-1α; ^##^ *p* < 0.05; H_2_O_2_-treated siCon vs. siNrf-2 in PGC-1α stable cells; ^†^ *p* < 0.05; H_2_O_2_-untreated siCon vs. siNrf-2 in PGC-1α cells.

We examined whether the anti-apoptotic and anti-oxidative effects on H_2_O_2_-treated PGC-1α cells were dependent on the Nrf-2 level. The level of activated caspase 3, which was reduced by PGC-1α overexpression, was partly restored by Nrf-2-specific reduction on H_2_O_2_-treated PGC-1α cells (Fig. 7A). In addition, the ROS level, which was reduced by PGC-1α overexpression, was also partly recovered by Nrf-2-specific reduction in H_2_O_2_-treated PGC-1α cells (Fig. 7B).

### Regulation of the p38/GSK3β/Nrf-2 axis by PGC-1α was involved in the cytoprotective effects of PGC-1α

**Figure 8:**
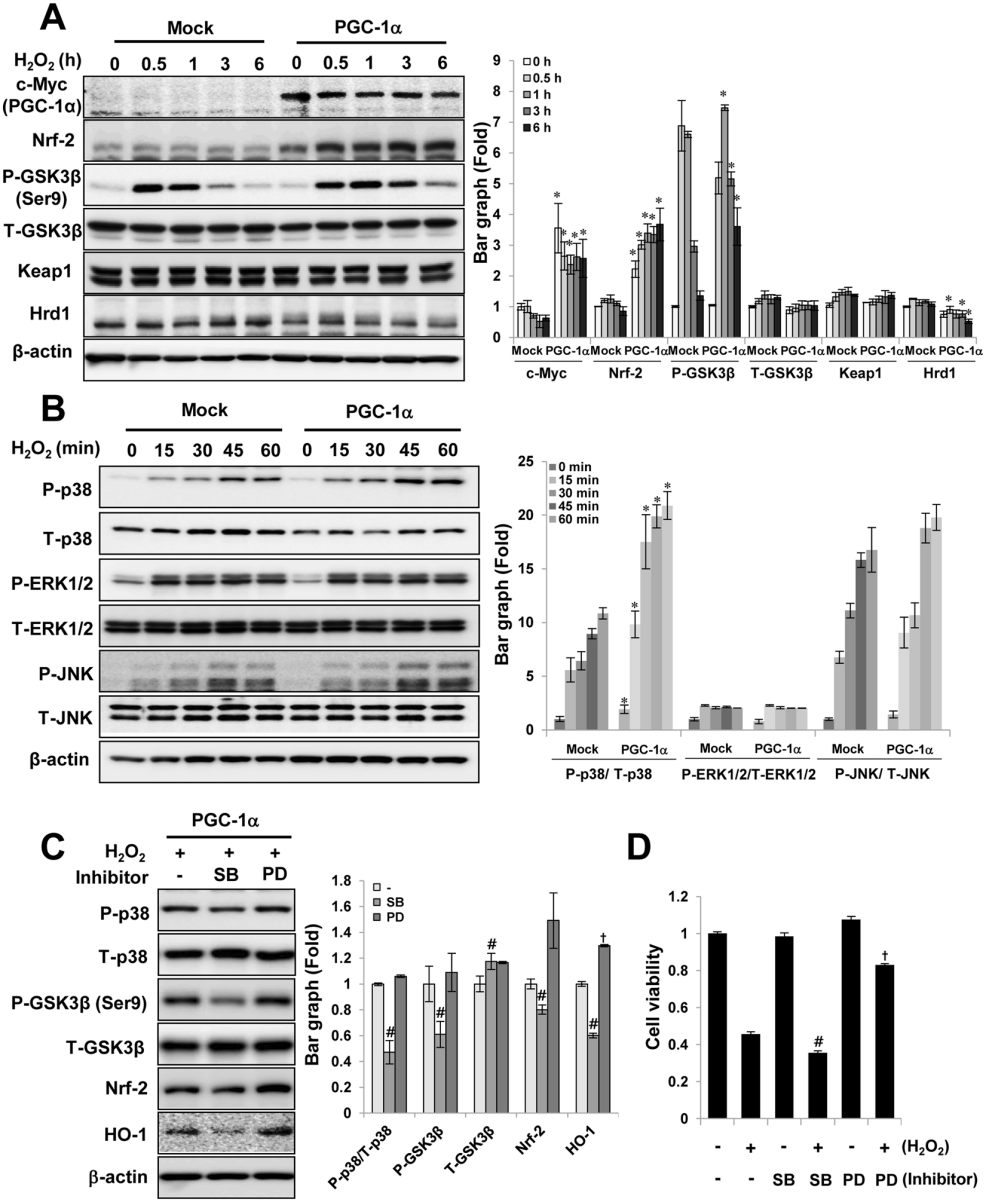
The involvement of p38/GSK3β/Nrf-2 axis for cytoprotective effects of PGC-1α. To understand the molecular mechanism for cytoprotective effects of PGC-1α, the activation of Keap1-, GSK3β- or Hrd1 (**A**) and MAPKs (**B**), as an upstream signal molecules of Nrf-2, were compared to Mock and PGC-1α cells for indicated time points after H_2_O_2_ treatment. To further elucidate the specificity of p38-mediated signal cascade for cytoprotective effects of PGC-1α cells, p38 specific effects were analyzed by western blotting (**C**) and MTT assay (**D**) in PGC-1α cells treated with H_2_O_2_ in the presence or absence of p38 inhibitor (SB203580) for 2 h or 4 h, respectively. Inhibitor of ERK1/2 (PD98059) was used as non-effective control on PGC-1α effect. Activation of p38 or inactivation of GSK3β was checked with phosphor specific antibody at Thr180/Tyr182 residue or at Ser9 residue, respectively. Full-length blots of each tested protein are reported in Supplementary Figure S7. Error bars denote the mean ± S.D. of triplicate samples. MTT assay was performed to n = 7. *p < 0.05 H_2_O_2_-untreated Mock vs. PGC-1α at indicated time points; ^#^ *p* < 0.05; p38 inhibitor treated vs. untreated; ^†^ *p* < 0.05; ERK1/2 inhibitor treated vs. untreated.

Three ubiquitin ligase systems that act as negative regulator of Nrf-2 have been reported, those are, the Keap1-, GSK3β-, and Hrd1-mediated systems^24, 33–35^. Nrf-2 binds with these negative regulators, and hence maintains Nrf-2 to basal level. Keap1 and GSK3β are present in cytoplasm, and Hrd1 in the endoplasmic reticulum (ER). We analyzed whether the molecular mechanisms underlying Nrf-2 upregulation in PGC-1α cells are Keap1-dependent or GSK3β-dependent or Hrd1-dependent. In cytosolic negative regulators of Nrf-2, The phosphor form of GSK3β at Ser9 was considerably increased in H_2_O_2-_treated PGC-1α cells at 1, 3, and 6 h as compared to the levels in Mock cells, whereas the Keap1 levels did not show a significant difference between the two groups (Fig. 8A). Hrd1, as a negative regulator of Nrf-2 in the endoplasmic reticulum, was also reduced in H_2_O_2-_treated PGC-1α cells as compared to the levels in Mock cells, although the difference in Hrd1 was lesser than that of GSK3β.

We investigated whether any of the other upstream molecules were involved in the GSK3β-inactivated Nrf-2 upregulation. Interestingly, p38, an upstream signaling molecule affected by GSK3β inactivation^36^, was specifically activated in H_2_O_2-_treated PGC-1α cells (Fig. 8B). p38 inactivation by treatment with a p38 inhibitor (SB203580, 5 μM) in H_2_O_2-_treated PGC-1α stable cells led to decreases in GSK3β inactivation, followed by decrease in Nrf2/HO-1 expression (Fig. 8C) and cell viability (Fig. 8D). In contrast, use of an ERK inhibitor (PD98059, 50 μM), used as a non-effective control on PGC-1α effect, did not lead to significant difference.

## Discussion

This study showed that PGC-1α is physiologically involved for the cytoprotective effects and one of its regulation mechanisms is the regulation of the p38/GSK3β/Nrf-2 axis by PGC-1α overexpression.

In the current study, we found decreased PGC-1α expression in I/R-induced AKI, which is associated with impaired renal function. The S3 segment of the proximal tubule and the thick ascending limb of Henle are highly susceptible to AKI, such as ischemic injury^37^. Moreover, an earlier study involving *in situ* hybridization for PGC-1α mRNA showed that PGC-1α is mainly expressed in proximal tubules and the thick ascending limb of Henle^16^. In addition, the PGC-1α protein level in H_2_O_2_-treated HK-2 cells was gradually decreased at high H_2_O_2_ concentrations or following longer exposures to H_2_O_2_. These findings are consistent with previous observations^38, 39^. And also, H_2_O_2_-induced PGC-1α downergulation was inhibited by NAC pre-treatment in H_2_O_2_-treated HK-2 cells. It has been recently reported that NAC plays a role as a mitochondrial enhancer as well as an antioxidant precursor to glutathione (GSH)^40^. In psychiatry and related neurodegenerative diseases, NAC used to increase mitochondrial resilience and prevent allostatic load by inhibiting mechanism of oxidative stress and inflammation^41, 42^. Given the prominent role of PGC-1α in mitochondrial biology, it is not surprising that PGC-1α is involved in the cellular response to ischemia. These findings suggest that PGC-1α could be a potential target to improve renal recovery following I/R-induced kidney injury. In stable cells, PGC-1α overexpression attenuated H_2_O_2_-induced cellular toxicity via anti-apoptotic and anti-oxidative effects. Mitochondria are the central executer of apoptosis^43^, and ROS generation has been suggested to be a major inducer of mitochondrial dysfunction and to play an important role in apoptosis regulation^44^. Our results suggest that a defect in PGC-1α is one of the major causes of H_2_O_2_-induced renal tubule cell apoptosis, and provides a novel strategy for preventing ROS-induced kidney tubule injury.

Nrf-2 serves as a master player of mitochondrial redox homeostasis by regulating the expression of diverse cytoprotective proteins that allow for cellular adaptation and survival under stress conditions^45, 46^. The findings of the current study suggest that the PGC-1α upregulated Nrf-2 expression and sequentially induced phase 2 detoxifying enzymes and related proteins, such as HO-1, leading to a cytoprotective effect against ROS-mediated injury. Consistent with our data, it has recently been reported that Nrf-2 knockout cells have higher mitochondrial ROS levels than wild-type cells, suggesting that Nrf-2 regulates mitochondrial ROS production^47^. In addition, HO-1-knockout mice were found to be markedly sensitized to diverse forms of AKI^48^. In this study, we didn’t check whether or not Nrf-2 mediated HO-1 expression in PGC-1α stable cells is specific in mitochondria. The several papers were reported about its mitochondrial function^49^. HO-1 overexpression appears to protect the heart from oxidative injury by regulating mitochondrial quality control^50^. In addition to HO-1, it has been reported that PGC-1α regulates the mitochondrial antioxidants, such as MnSOD, Prx5, and Prx3 in vascular endothelial cells^51^. In agreement with previous data, we also identified upregulation of their expression in PGC-1α stable cells (data not shown). But, whether upregulation of their expression in PGC-1α stable cells was dependent on the expression of Nrf-2 would be further elucidated.

In this study, we first demonstrated that regulation of the p38/GSK3β/Nrf-2 axis by PGC-1α is one of mechanisms protecting renal tubule cells against ROS-mediated cellular toxicity. Under basal condition, Nrf-2 is a short-lived protein that is subjected to continuous ubiquitination and proteasomal degradation. Under stress condition, Keap1 is inactivated by oxidation of the reactive cysteine residue or down-regulated by epigenetic silencing^52^; then, GSK3β is inactivated by Ser9 phosphorylation. Nrf-2/Keap1 or Nrf-2/GSK3β complex is disrupted by conformational change. Consequently, Nrf-2 stabilizes and is then translocated to the nucleus for Nrf-2 mediated gene expression. Among cytosolic negative regulators of Nrf-2, we showed that GSK3β, in particular, changed to the inactive form on phosphorylation at Ser9 in PGC-1α cells at the increased time point of H_2_O_2_ treatment, while Keap1 did not. Consistent with our data, mice with renal-proximal-tubule-specific GSK-3β knockout and chemical inhibition of GSK3 were found to have better survival and renal function than wild-type mice in an HgCl_2_-induced model of AKI in a previous study^53^. In our study, the protein expression of Nrf-2 was increased both in cytosol and in nucleus by the overexpression of PGC-1α. The degree of expression was greater in cytosolic fractionation than in nuclear fraction. It is speculated that Nrf-2 is more regulated by cytosolic events, such as inactivation of GSK3β. However, detailed mechanisms of Hrd1-dependent Nrf-2 upregulation would be further elucidated. Furthermore, our data showed that p38 was specifically activated in PGC-1α stable cells and that p38-specific inactivation in PGC-1α cells inversely affected the sequential activation and expression of GSK3β/Nrf-2/HO-1 as its downstream targets. 15d-PGJ(2), a potent endogenous ligand for peroxisome proliferators-activated receptor gamma, induces HO-1 expression using p38 MAP kinase and Nrf-2 pathway in ROS-mediated VSMCs; an inhibitor of p38 MAP kinase was found to abolish 5d-PGJ(2)-induced HO-1 expression^54^. Although we didn’t check the regulation mechanism of p38/GSK3β/Nrf-2 axis by PGC-1α in I/R-injured kidney, several studies reported that the reduction of Nrf-2 and its downstream molecule, HO-1, in I/R-injured kidney resulted in I/R-induced ROS generation and inflammation, followed by acute kidney injury^55, 56^. Renal ischemia-reperfusion injury (IRI) is a major cause of AKI, which has common pathophysiological features including renal tubular apoptosis/necrosis, ROS generation, mitochondria dysfuncrion and inflammatory cell infiltration. Therefore, it is reasonable to explore and to understand the therapeutic mechanism to treat common pathophysiology features of IRI. Our studies imply that PGC-1α may be one of therapeutic targets against AKI.

In conclusion, PGC-1α protects human renal tubule cells from H_2_O_2_-mediated apoptotic injury by upregulating Nrf-2 via GSK3β inactivation mediated by activated p38. Regulation of the p38/GSK3β/Nrf-2 axis by PGC-1α could be a viable target for ameliorating mitochondrial dysfunction following AKI.

## Materials and Methods

### Materials

The selective antibiotics, zeocin was purchased from Invitrogen (CA, USA). 5-(and-6)-chloromethyl-2′,7′-dichlorodihydrofluorescein diacetate, acetyl ester (CM-H2DCFDA) and MitoTracker were purchased from Molecular Probes (Invitrogen, CA, USA). Antibodies against PGC-1α, Keap1, and Nrf-2, were purchased from Santa Cruz (Dallas, Texas, USA). Antibody against Hrd1 was purchased from Novus Biologicals (Littleton, CO, USA). Antibodies against caspase 3, cleaved caspase 3, Bax, Bcl2, phosphor-p53, total-p53, phosphor-GSK3β, totoal-GSK3β, phosphor-p38, phosphor-ERK1/2, phosphor-JNK, total-p38, total-ERK1/2, total-JNK, HO-1 and c-myc were all purchased from Cell Signaling Technology (Danvers, MA, USA). SB203580 and PD98059 were purchased from Calbiochem (Cat#559398 for SB203580 and Cat#513001 for PD98059, Darmstadt, Germany). N-acetyl-L-cysteine (NAC, Cat#A7250) and Thiazolyl Blue Tetrazolium (Cat#M2128) for MTT assay was purchased from sigma-aldrich. For over-expression of human PGC-1α in HK-2 cells, human *PGC-1α/pCDNA4* plasmid was purchased from Addgene (Cat#10974, Cambridge, USA). Nrf-2 specific siRNA (Cat# sc-37030) and control siRNA (Cat# sc-37007) were purchased from purchased from Santa Cruz (Dallas, Texas, USA). DhamaFECT 1 Transfection reagent was purchase from GE Healthcare (Cat# T-2001-02, USA).

### Animals

All methods were carried out in accordance with relevant guidelines and regulations.

All experimental protocols were approved by the Animal Care Regulations (ACR) Committee of Chonnam National University Medical School (CNU IACUC-H-2016-26). Eight-week-old male C57BL6 mice were purchased by Samtako (Korea). Mice were anesthetized with 2% isofurane and 100% oxygen and placed on a temperature-regulated table (38.5 °C) to maintain body temperature. Renal ischemia was induced by clamping both renal pedicles with micro clamp (ROBOZ, Gaithersburg, USA) for 30 min. I/R group (n = 4) was sacrificed after 1 day of reperfusion. Control group (n = 4) underwent the same procedure, except that the clamp was not applied. Blood samples were then collected from the heart, and the left kidney was rapidly removed and processed for western blotting or fixed in 4% paraformaldehyde solution for immunohistochemistry (IHC). The right kidney was frozen at −80 °C for real-time PCR.

### Cell culture

HK-2 cells (ATCC, Manassas, VA), were cultured in complete DMEM-F12 media (WelGene, Daegu, Korea) supplemented with 10% fetal bovine serum, 50 U/ml penicillin and 50 μg/ml streptomycin at 37 °C under a humidified 5% CO_2_ atmosphere. Stably transfected HK-2 cells ectopically expressing Mock and PGC-1α constructs were maintained in complete medium containing 200 µg/ml of zeocin (Invitrogen, Carlsbad, CA, USA).

### Stable cell lines

HK-2 cells were then transfected with 2 μg of empty vector (Mock) or *PGC-1α* DNA using 6 μl of Fusion HD reagent (Promega, Madison, WI, USA) in antibiotic-free DMEM-F12. Beginning 1 day after transfection, transfectants were selected in DMEM-F12 containing 200 μg/ml zeocin, which was refreshed every 3 days for 2 weeks. Colonies surviving in the selection medium were collected and sequentially plated in 48, 12, 6-well plates, and then 60 and 100 mm dishes. Cells stably overexpressing human PGC-1α were identified by immunoblotting with anti c-myc and anti β-actin antibodies or by PCR analysis using zeocin primers 5′-ATGGCCAAGTTGACCAGTGCCGTT-3′ (forward) and 5′-GTCCTGGTCCTCGGCCACGAAGTG-3′ (reverse)^57^. A loading control was analyzed by using GAPDH primers 5′-ACCACAGTCCATGCCATCAC-3′ (forward) and 5′-TCCACCACCCTGTTGCTGT-3′ (reverse).

### Tunel staining in kidney tissue

Apoptosis was determined using the ApopTag Plus Peroxidase *In Situ* Apoptosis Detection Kit (Chemicon International; Temecula, CA, USA) according to the manufacturer’s protocol. The sections were counterstained with hematoxylin and examined by light microscopy. Image was magnified at x100.

### siRNA knockdown

RNA interference of Nrf-2 was performed using an Nrf-2-specific siRNA from Santa Cruz (Cat# sc-37030). Briefly, cells were transfected with indicated concentration of siRNA (30 nM and 50 nM) using DhamaFECT 1 transfection reagent according to the manufacturer’s protocol. Cells transfected with control siRNA (Santa Cruz, Cat# sc-37007) were used as controls for direct comparison.

### RT-PCR & real-time PCR

To quantify mRNA levels, total RNA was extracted from frozen mouse kidney or HK-2 cells using TRIzol reagent (Invitrogen). cDNA was then reverse transcribed from 1 μg samples of total RNA using QuantiTect Reverse Transcription kit (Qiagen Science, Maryland, USA). Real-time PCR was performed using QuantiTect SYBR Green PCR master mix (Qiagen Science, Maryland, USA) and a Rotor-Gene TM 3000 Detector System (Corbette research, Mortlake, New South Wales, Australia). RT-PCR primer sequences were as follows: for mouse β-actin, 5′-ATATCGCTGCGCTGGTCGTC-3′ (F) and 5′-GATGGGCACAGTGTGGGTGA-3′ (R); for mouse PGC-1α, 5′-AATGCAGCGGTCTTAGCACT-3′ (F) and 5′-TTTCTGTGGGTTTGGTGTGA-3′ (R). The primer sequences used for real time-PCR were as follows: for human GAPDH, 5′-GACATCAAGAAGGTGGTGAA-3′ (F) and 5′-TGTCATACCAGGAAATGAGC-3′; for human PGC-1α, 5′-TCTCAGTACCCAGAACCATGCA-3′ (F) and 5′-GCTCCATGAATTCTCAGTCTTAACAA-3′ (R); for Nrf-2, 5′-GAGAGCCCAGTCTTCATTGC-3′ (F) and 5′-TGCTCAATGTCCTGTTGCAT-3′ (R). Data from the reaction were collected and analyzed with the appropriate software package from Corbett Research.

### Cell viability

The MTT assay was applied to test cell viability. In brief, stable HK-2 cells (Mock or PGC-1α) were seeded into plates at 1 × 10^4^ cells per 96 wells. After 1 day, cells were incubated in 100 μl of 0.5 mM H_2_O_2_ diluted in HBSS for the indicated time at 37 °C and with 5% CO_2_. Subsequently, 10 μl of MTT reagent (5 mg/ml) was added to yield a final concentration of 0.5 mg/ml. After 2 h of additional incubation, all solution was removed, and 100 μl of DMSO was directly added to the cells to dissolve water-insoluble MTT-formazan. Absorption at 590 nm was determined with an ELISA reader (BioTek, Winooski, VT, USA).

### Apoptosis assay

The number of apoptotic cells was quantified using the Ezway Annexin V-FITC Apoptosis Detection Kit (KOMA BIOTECH, Seoul, Korea) according to the manufacturer’s protocol. Cells were sequentially probed with Annexin V-FITC and propidium iodide (PI) dye. Fluorescent intensity was measured by a FACSCalibur^TM^ flow cytometry (BD Biosciences, San Jones, CA, USA).

### DAPI staining for apoptosis analysis

The apoptotic effect was analyzed by using florescent nuclear dye 4′,6-diamidino-2-phenylindole dihydrochloride (DAPI). The cells were seeded onto four well-cell culture slides (5 × 10^4^/well) and treated as mentioned previous. Cells were then washed with PBS and fixed in 4% paraformaldehyde for 10 min. Subsequently the cells were permeablized with equilibration buffer (1% BSA and 0.5% Triton X-100 in PBS) and stained with DAPI dye. After staining, the images were captured using a confocal microscope (LSM 510; Carl Zeiss). Image was magnified at x800, Bar = 20 μm.

### Measurement of ROS generation

Level of intracellular and mitochondrial ROS^58, 59^ were assessed using 5,6-chloromethyl-2′,7′-dichlorodihydrofluorescein diacetate (CM-H_2_DCFDA; Invitrogen, Carlsbad, CA, USA) or Rosamine-based MitoTracker probes (MitoTracker Red CM-H_2_XROS, Invitrogen, Carlsbad, CA, USA), respectively. Labeling with both probes was conducted on lived cells, but not fixed cells. After cells were treated with 0.5 mM H_2_O_2_ for 30 min, and then were loaded with 10 μM CM-H_2_DCFH-DA or 0.2 μM CM-H_2_XROS for 30 min at 37 °C. Images were immediately visualized using confocal microscopy on a laser scanning microscope (LSM 510; Carl Zeiss), and analyzed using imageJ software. Image was magnified at x200 or x400.

### Preparation of nuclear and cytoplasmic extracts

HK-2 cells were lysed using NE-PER® nuclear extraction reagent (NER) (Pierce Biotechnology, Rockford, IL, USA) according to the manufacturer’s protocol. Briefly, 100 µL of ice-cold cytoplasmic extraction reagent (CER) I was added to the harvested cells. After incubated on ice for 10 min, ice-cold CER II was added to the tube. The tube was centrifuged at 16,000 × *g* for 5 min and the supernatant fraction was saved as cytosolic protein. The remained pellet was suspended in 50 µL of ice-cold NER. After centrifuging the tube at 16,000 × *g* for 10 min, the supernatant (nuclear protein) fraction was transferred to a clean tube.

### Statistical analysis

All experiments were conducted in triplicate. The results were expressed as mean ± standard deviation (S.D). We used Student’s t test for the comparison between two gorups, and used One-way ANOVA when we compared more than two groups. Differences with values of *p* < 0.05 were considered significant.

## Electronic supplementary material

Supplementary Dataset
