# Supplementary material for: PGC-1α attenuates hydrogen peroxide-induced apoptotic cell death by upregulating Nrf-2 via GSK3β inactivation mediated by activated p38 in HK-2 Cells
Source: Sci Rep. 2017 Jun 28;7:4319. doi: 10.1038/s41598-017-04593-w (PMC5489530; doi:10.1038/s41598-017-04593-w)
Supplement: Supplementary file 1 — Supplementary Dataset [file 41598_2017_4593_MOESM1_ESM.doc]

**Supplementary Information**

**PGC-1α attenuates hydrogen peroxide-induced apoptotic cell death by upregulating Nrf-2** **via GSK3β inactivation mediated by activated p38 in HK-2 Cells**

**Hoon-In Choi, Hye-Jin Kim**, **Jung-Sun Park, In-Jin Kim, Eun Hui Bae, Seong Kwon Ma, Soo Wan Kim^*^**

*Department of Internal Medicine, Chonnam National University Medical School, Gwangju, Republic of Korea*

^*^Correspondence:

Soo Wan Kim, MD, PhD.

Department of Internal Medicine,

Chonnam National University Medical School,

42 Jebongro, Gwangju 61469, Republic of Korea.

Telephone number: +82-62-220-6271,

Fax number: 82-62-225-8578,

E-mail: [skimw@chonnam.ac.kr](mailto:skimw@chonnam.ac.kr)

**Supplementary Figure 1.** Uncropped image of gels and blots included in Fig.1. The red boxes indicates the cropped regions.

**Supplementary Figure 2.** Uncropped image of western blots included in Fig.2. The red boxes indicates the cropped regions.

**Supplementary Figure 3.** Uncropped image of gels and blots included in Fig.3. The red boxes indicates the cropped regions.

**Supplementary Figure 4.** Uncropped image of gels and blots included in Fig.4. The red boxes indicates the cropped regions.

**Supplementary Figure 5.** Uncropped image of gels and blots included in Fig.6. The red boxes indicates the cropped regions.

**Supplementary Figure 6.** Uncropped image of gels and blots included in Fig.7. The red boxes indicates the cropped regions.

**Supplementary Figure 7.** Uncropped image of gels and blots included in Fig.8. The red boxes indicates the cropped regions.
